# A Study of Driver's Route Choice Behavior Based on Evolutionary Game Theory

**DOI:** 10.1155/2014/124716

**Published:** 2014-12-29

**Authors:** Xiaowei Jiang, Yanjie Ji, Muqing Du, Wei Deng

**Affiliations:** ^1^School of Transportation, Southeast University, 35 Jinxianghe Road, Nanjing, Jiangsu 210096, China; ^2^College of Civil and Transportation Engineering, Hohai University, 1 Xikang Road, Nanjing, Jiangsu 210098, China

## Abstract

This paper proposes a route choice analytic method that embeds cumulative prospect theory in evolutionary game theory to analyze how the drivers adjust their route choice behaviors under the influence of the traffic information. A simulated network with two alternative routes and one variable message sign is built to illustrate the analytic method. We assume that the drivers in the transportation system are bounded rational, and the traffic information they receive is incomplete. An evolutionary game model is constructed to describe the evolutionary process of the drivers' route choice decision-making behaviors. Here we conclude that the traffic information plays an important role in the route choice behavior. The driver's route decision-making process develops towards different evolutionary stable states in accordance with different transportation situations. The analysis results also demonstrate that employing cumulative prospect theory and evolutionary game theory to study the driver's route choice behavior is effective. This analytic method provides an academic support and suggestion for the traffic guidance system, and may optimize the travel efficiency to a certain extent.

## 1. Introduction

In recent years, with the rapid development of information technology, traffic information system has had a great effect on travel decision-making behavior. Drivers may respond to the information through adjusting the travel mode, destination, departure time, and speed, but most commonly by altering routes [1–5]. The aim of this work is to propose such an analytic method that is able to take traffic information into account to explore the mechanism of route choice behavior.

Researches related to route choice have been conducted in many perspectives. Chen and Jovanis [6] and Polydoropoulou et al. [7] claimed that drivers' attitudes towards communication, technology, and transportation system reliability affected their route decision-making process. Jan et al. [8], Li et al. [9], and Srinivasan and Mahmassani [10] found that the ultimate route choice decision was inherently a multiple-objective behavior. They considered many factors other than the conventional measurement variables and demonstrated that the factors had a major impact on route decision-making process. Bogers et al. [11] and Ben-Elia et al. [12] constructed simulation experiments to explore the influences of information, learning, and habit on choices between two routes. Chorus et al. [13] presented a discrete choice model to research driver's responses to VMS. The model indicated that the preferences and beliefs had significant impacts on driver's choice behavior. Ben-Elia and Shiftan [14] conducted a laboratory controlled experiment to model the route choice behavior when information was provided in real time. The results showed that information and previous travel experiences had a combined effect on driver's route choice behavior. Kusakabe et al. [15] conducted a SP survey to investigate the effects of traffic incident information provided on VMS on driver's route choice behavior. The results showed that drivers assumed the travel time of their alternative routes according to the incident information of the road section provided by VMS. Ben-Elia et al. [16] conducted a route choice experiment to investigate the impact of the accuracy of traffic information on route choice. The results suggested that decreasing accuracy shifted choices mainly from the risk to the reliable route but also to the useless alternative.

The above researchers studied the route choice behavior in the perspective of expected utility theory (EUT) [17] or random utility theory (RUT) [18–21]; little work has been done from the point of bounded rational. Drivers evaluate the alternative routes by individual experience, cognition, and attitudes which are not considered in the EUT and RUT models. Hence, many alternative theories are proposed, for example, prospect theory (PT) [22], cumulative prospect theory (CPT) [23], rank-dependent expected theory [24], regret theory [25], and behavioral portfolio theory [26]. Among them, CPT describes the bounded rational behaviors under risk and uncertainty preferably, so it draws the most attention.

Looking at the issue from another point, route choice is a dynamic selection process because of the real-time traffic information and the updated road condition. Little work has been done from the point of dynamic selection process to discuss how drivers make route choice decisions considering traffic information. Evolutionary game theory is the theory that discusses system's dynamic evolution process under bounded rational conditions.

The purpose of this paper is to describe how drivers adjust their route choice behaviors under the influence of traffic information from a bounded rational and dynamic selection process perspective. The remainder of the paper is organized as follows.  Section 2 describes the basic theories applied in this paper, including cumulative prospect theory and evolutionary game theory. In Section 3, a network with two alternative routes is constructed to model the drivers' route choice behaviors and the route choice model derived from CPT is established. The analysis of the equilibrium network state is given in the following. Limitations of the proposed modeling method and the further research directions are discussed in Section 4.

## 2. Theory Preliminaries

### 2.1. Cumulative Prospect Theory

Cumulative prospect theory (CPT) is a method for descripting decisions under risk and crisis which was introduced by Tversky and Kahneman in 1992. CPT distinguishes the choice process into two phases: framing and valuation. In the phase of framing, the decision maker constructs a representation of the acts, contingencies, and outcomes that are relevant to the decision. In the phase of valuation, the decision maker assesses the representation value of each prospect and chooses the largest one accordingly [23].

The main opinion of CPT is that people tend to think of possible outcomes relative to a certain reference point rather than to the final status, a phenomenon which is called framing effect. Moreover, they have different risk attitudes towards gains (i.e., outcomes above the reference point) and losses (i.e., outcomes below the reference point) and care generally more about the potential losses than the potential gains. Finally, people usually overweigh the extreme, but unlikely, events, however, underweigh the “average” events.

**Figure 1:**
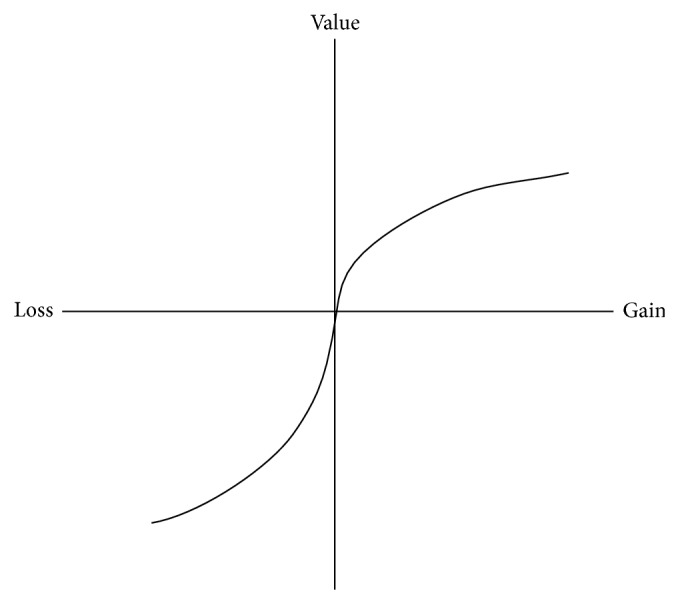
Shape of value function.

**Figure 2:**
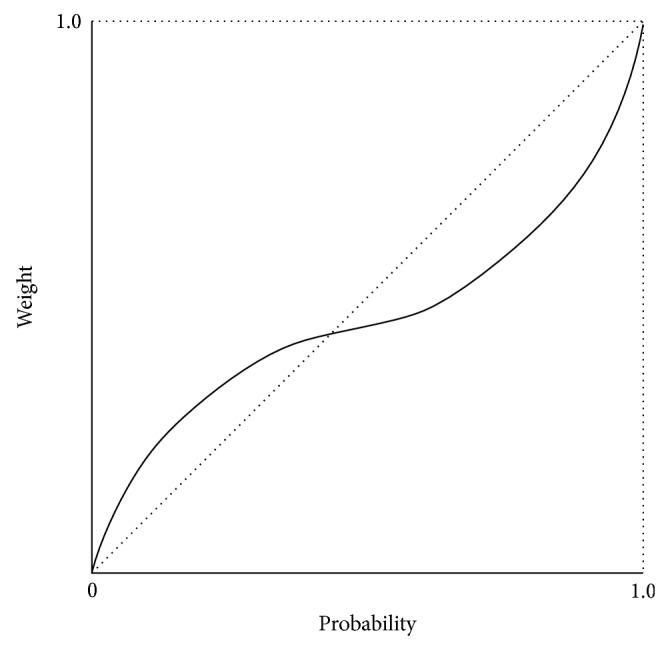
Shape of weighting function.

CPT incorporates these opinions in a modification of the expected utility theory by replacing the final wealth with the payoffs relative to the reference point, replacing the utility function with the value function that depends on the relative payoffs, and replacing the cumulative probabilities with the weighting cumulative probabilities. The subjective utility of a risky outcome is described by a probability measure *p*:

(1) Up=∫−∞0vxddxwFxdx +∫0+∞vxddx−w1−Fxdx,

where *v*(*x*) is the value function (typical form shown in Figure 1) and *w*(*x*) is the weighting function (Figure 2) and *F*(*x*) = ∫_−*∞*_^*x*^ *dp*.

#### 2.1.1. Value Function

The value function proposed by Tversky and Kahneman [23] is employed in our work. It is expressed as follows:

(2) $$\begin{array}{c} v\left(x\right)=\left\{\begin{array}{cc} x^{\alpha }, & \text{if}\;\;x\geq 0,\\ -\lambda {\left(-x\right)}^{\beta }, & \text{if}\;\;x<0, \end{array}\right. \end{array}$$

where *x* is the outcome relative to a certain reference point. *α* and *β* are the estimation coefficients which determine the convexity or concavity of the value function shape. *λ* is the loss aversion coefficient. Both *α* and *β* fall between 0 and 1; particularly, *α* = *β* = 1 represents the pure loss aversion. *λ* should be larger than 1 to describe the degree of loss aversion and to resemble the S-shape in Figure 1.

It is apparent from Figure 1 that the value function is convex above the reference point (*v*′′(*x*) ≤ 0, *x* ≥ 0) and concave below the reference point (*v*′′(*x*) ≥ 0, *x* ≤ 0). It is steeper for losses than for gains (*v*′(*x*) < *v*′(−*x*) for *x* ≥ 0).

#### 2.1.2. Weighting Function

Based on the research of Tversky and Kahneman [23], the weighting function is defined by two inversely S-shaped formulations:

(3) $$\begin{array}{c} w^{+}\left(p\right)=\frac{p^{\gamma }}{{\left(p^{\gamma }+{\left(1-p\right)}^{\gamma }\right)}^{1/\gamma }},\\ w^{-}\left(p\right)=\frac{p^{\delta }}{{\left(p^{\delta }+{\left(1-p\right)}^{\delta }\right)}^{1/\delta }}, \end{array}$$

where *w* ^+^ and *w* ^−^ represent the weighting function for gains and losses, respectively. *γ* and *δ* indicate the level of distortion in probability judgment and they should fall between 0 and 1. Decreasing *γ* and *δ* causes the shape of the weighting function to become more curved and to cross the 45-degree line farther to the right. Figure 2 presents the shape of weighting function.

*w* ^+^ and *w* ^−^ are strictly increasing functions from the unit interval into itself satisfying *w* ^+^(0) = *w* ^−^(0) = 0 and *w* ^+^(1) = *w* ^−^(1) = 1 [27].

#### 2.1.3. Cumulative Prospect Value

Based on CPT, the representation value of a prospect *f* is represented as follows:

(4) $$\begin{array}{c} V\left(f\right)=V\left(f^{+}\right)+V\left(f^{-}\right), \end{array}$$

where *V*(*f* ^+^) is the cumulative value of the prospect gains and *V*(*f* ^−^) is the cumulative value of the prospect losses. *V*(*f*) is the function of decision weights *π*_*i*_ and value function *v*(*x*_*i*_) and it is defined as follows:

(5) $$\begin{array}{c} V\left(f^{+}\right)=\overset{n}{\underset{i=0}{\sum }}\pi_{i}^{+}v\left(x_{i}\right),\;\;V\left(f^{-}\right)=\overset{0}{\underset{i=-m}{\sum }}\pi_{i}^{-}v\left(x_{i}\right). \end{array}$$

*π* ^+^(*f* ^+^) = (*π*_0_^+^,…, *π*_*n*_^+^) is the decision weight of the gains and *π* ^−^(*f* ^−^) = (*π*_−*m*_^−^,…, *π*_0_^−^) is the decision weight of the losses. The decision weights are further defined by

(6) $$\begin{array}{c} \pi_{n}^{+}=w^{+}\left(p_{n}\right),\;\;\pi_{-m}^{-}=w^{-}\left(p_{-m}\right), \end{array}$$

(7) πi+=w+pi+⋯+pn−w+pi+1+⋯+pn,0≤i≤n−1,

(8) πi−=w−p−m+⋯+pi−w−p−m+⋯+pi−1,1−m≤i≤0.

### 2.2. Evolutionary Game Theory

Evolutionary game theory (EGT) is a theory that combines game theory with dynamic evolution process analysis. EGT is useful in this context by defining a framework of contests, strategies, and analytics into which Darwinian competition can be modelled. EGT originated in 1973 with Smith and Price's formulization of the way in which such contests can be analyzed as “strategies” and the mathematical criteria that can be used to predict the resulting prevalence of such competing strategies [28]. EGT differs from the classical game theory by focusing more on the dynamics of strategy change which is influenced not solely by the quality of the various competing strategies, but also by the effect of the frequency with which those various competing strategies are found in the population.

#### 2.2.1. Evolutionary Stable Strategy

Evolutionary stable strategy (ESS) was defined and introduced by Smith and Price in a 1973 Nature paper [28]. An ESS is a strategy which, if adopted by a population in a given environment, cannot be invaded by any alternative strategy that is initially rare. The “evolutionarily” stable is a Nash equilibrium solution; once it is fixed in a population, natural selection alone is sufficient to prevent alternative strategies from invading successfully.

ESS presumes that individuals have no control over their strategies and need not be aware of the game. To be an ESS, a strategy must be resistant to alternatives. Every ESS corresponds to a Nash equilibrium solution, but not all Nash equilibrium solutions are ESSes.

The mathematical definition of ESS can be expressed as follows. For a very small positive *ε*, every *σ* ≠ *σ* ^*^ meets the following condition:

(9) $$\begin{array}{c} \mu \left(\sigma^{*},\left(1-\varepsilon \right)\sigma^{*}+\varepsilon \sigma \right)>\mu \left(\sigma ,\left(1-\varepsilon \right)\sigma^{*}+\varepsilon \sigma \right). \end{array}$$

That is to say, for a small proportion *ε* of mutation behavior *σ* in population, taking strategy *σ* ^*^ will get higher utility, and the stable state as a result of strategy *σ* ^*^ cannot be invaded by a small mutation. Then the strategy *σ* ^*^ is the ESS. It is noteworthy that the mutational strategy is the strategy which is different from the strategy sets.

There are two properties for a strategy *σ* ^*^ to be an ESS. For all *σ* ≠ *σ* ^*^,

(10) $$\begin{array}{c} \mu \left(\sigma^{*},\sigma^{*}\right)>\mu \left(\sigma ,\sigma^{*}\right)\\ \text{or}\;\;\mu \left(\sigma^{*},\sigma^{*}\right)=\mu \left(\sigma ,\sigma^{*}\right),\;\;\mu \left(\sigma^{*},\sigma \right)>\mu \left(\sigma ,\sigma \right). \end{array}$$

The first property is called a strict Nash equilibrium solution. The second property means that although strategy *σ* is neutral with respect to the payoff against strategy *σ* ^*^, the population of players who continue to play strategy *σ* ^*^ has an advantage when playing against *σ*.

The limitation of ESS is that it is a static equilibrium without considering the dynamic evolutionary process. The stability equilibrium of evolution should be associated with the specific evolutionary process.

#### 2.2.2. Replicator Equations

The common methodology to study the evolutionary process is through the selection dynamics. It shows the growth rate of the proportion of people using a certain strategy. The basic expression of the selection dynamics is presented as

(11) $$\begin{array}{c} {\dot{\theta }}_{i}\left(t\right)=\theta_{i}\left(t\right)\cdot g_{i}\left(\theta \right), \end{array}$$

where *θ*_*i*_(*t*) is the proportion of the people who choose strategy *i* at time *t*, *g*_*i*_(*θ*) represents the specific selection process, and different learning mechanisms correspond to different function forms. The primary characteristic of the selection dynamics is that the pure strategy taken by no one in the initial state will never be used. Participants can only imitate the existing strategies; that is, the strategies did not reflect the mutation. This feature can be expressed in mathematics as follows:

(12) $$\begin{array}{c} \theta_{i}\left(t\right)⟶0⟹{\dot{\theta }}_{i}\left(t\right)⟶0. \end{array}$$

Among all kinds of game dynamic schemes, replicator dynamics (RD) by Taylor and Jonker [29] is most widely researched and a lot of relative conclusions have been obtained. The replicator dynamics is presented as

(13) $$\begin{array}{c} {\dot{\theta }}_{i}\left(t\right)=\theta_{i}\left(t\right)\cdot \left[\mu_{t}\left(s_{i}\right)-{\overset{-}{\mu }}_{t}\right],\;\overset{-}{\mu }=\overset{n}{\underset{i=1}{\sum }}\theta_{i}\left(t\right)\mu_{t}\left(s_{i}\right). \end{array}$$

In RD, each participant is on behalf of one kind of group with a uniform population distribution and the participants insist on taking a pure strategy *s*_*i*_. The growth rate *dθ*_*i*_/*dt* of the proportion *θ*_*i*_ taking the pure strategy is the strictly increasing function of the difference between the payoff *μ*(*s*_*i*_) and the average payoff  $\overset{-}{\mu }(t)$.

## 3. Route Choice Model Formulation and Dynamic Evolutionary Analysis

### 3.1. The Relationship between CPT and EGT

Cumulative prospect theory and evolutionary game theory deal with bounded rationality from two different perspectives: the former tries to handle individual irrationality from the perspective of psychological perception, while the latter focuses on the limited rationality in selection and decision [30]. The research results of CPT reveal the fact that people tended to magnify small probabilities and to minify large probabilities and they are more sensitive to losses than to gains of the same quantity. Evolutionary game theory interprets the mechanism that players are programmed to follow a certain choice scheme to behave or react according to the current system state. The process of looking for participants' strategies is the main point of evolutionary game theory as a kind of theory that researches the laws of decision.

### 3.2. Route Choice Model Formulation

**Figure 3:**
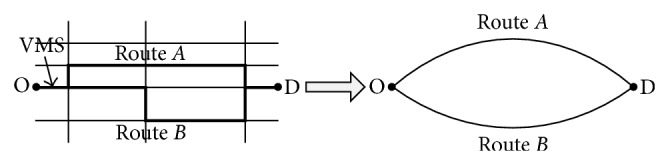
An example of two-route network.

**Figure 4:**
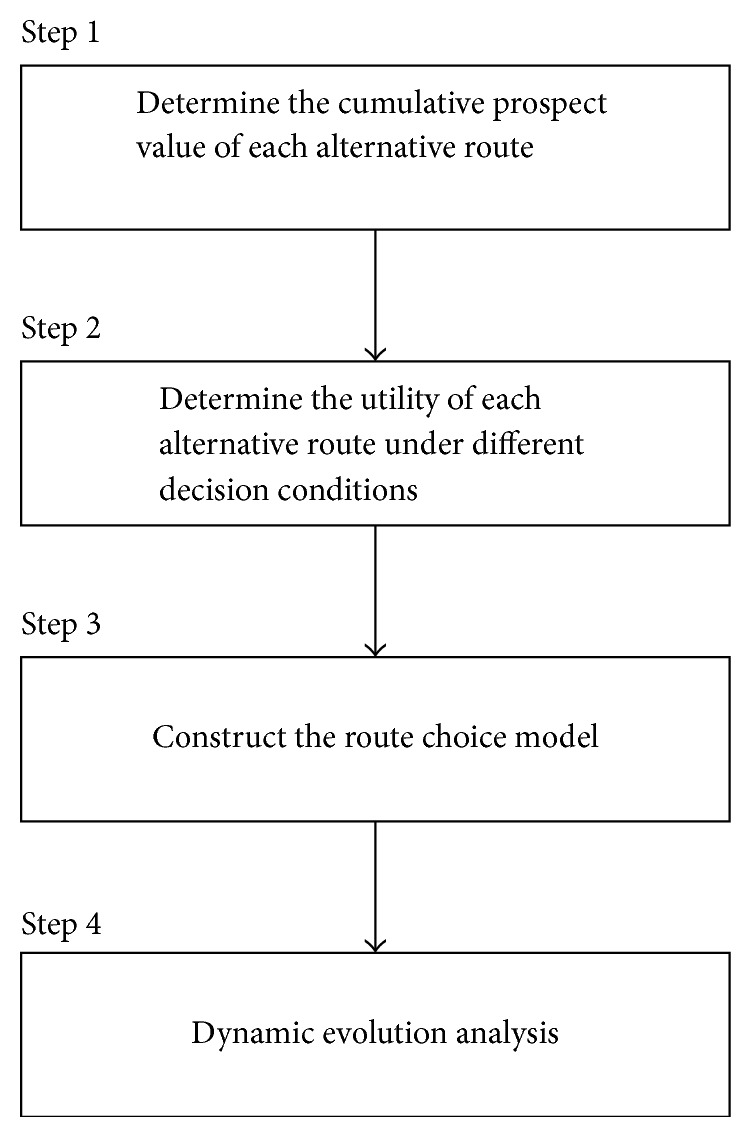
Flow chart of route choice modeling process.

In this section, we will take a two-route network, for example, to illustrate the route choice modeling process. The network consisted of route *A* and route *B*. Route *A* is the shortest route and route *B* is the recommended route provided by VMS when there are congestions in route *A*. The length of route *B* is longer than route *A*, and there is detouring distance when switching from route *A* to route *B*. The VMS is installed near the “O” point to display the real-time traffic information (see Figure 3). We assume that there are two types of drivers' distributions in this network. The first type prefers the shortest route as their route choice decisions. We call these drivers rigid demand drivers. The other type is prone to switching to the recommended route, and we call these drivers flexible demand drivers. Under the influence of traffic information, all drivers condition their route choice decisions on their perceptive travel time (payoff) of each possible route. The flow chart of the route choice modeling process is exhibited as Figure 4.

**Step 1 (determine the cumulative prospect value of each alternative route).**

For a specific road network, drivers determine the perceptive time of each alternative route based on their previous travel experiences. The travel time distribution of each alternative route is assumed to be identical and independent of each other. According to the central-limit theorem, the distribution of the perceived travel time of the alternative route approximately obeys the normal distribution. The distribution of the perceptive travel time is written as follows:

(14) $$\begin{array}{c} T_{k}\sim N\left(t_{k},{\left(\sigma_{k}\right)}^{2}\right),\;t_{k}=\frac{1}{n}\overset{n}{\underset{i=1}{\sum }}t_{i},\\ \sigma_{k}^{2}=\frac{1}{n-1}\overset{n-1}{\underset{i=1}{\sum }}{\left(t_{i}-t_{k}\right)}^{2}, \end{array}$$

where *T*_*k*_ is the perceived travel time of route *k*; *t*_*k*_ is the average travel time of route *k*; *t*_*i*_ is the travel time route *k*; *σ*_*k*_^2^ is the travel time variance of route *k*; *n* is the number of travels.

Because the free flow time can reflect the physical properties of the route in a certain extent, the reference point in this research is defined as the average value of the free flow time of all alternative routes. The reference point is represented as follows:

(15) $$\begin{array}{c} T_{0}=\frac{1}{K}\overset{K}{\underset{k=1}{\sum }}T_{k}^{\text{free}}, \end{array}$$

where *T*_0_ is the reference point; *T*_*k*_^free^ is the free flow time of route *k*; *K* is the number of the alternative route. In this network, *K* = 2.

Based on CPT, we assign each route *k* a value *V*(*k*). The *V*(*k*) is the cumulative prospect value. The value function of two alternative routes can be obtained based on (2). It is worth noting that *x* in (2) is expressed as *T*_0_ − *T*_*k*_ in our research. Based on the probability of each *x*, the weighting function can be obtained by (3). Thus, the cumulative prospect values of the two routes *V*(*A*) and *V*(*B*) are calculated by (4) to (8), respectively.

**Step 2 (determine the payoff under different decision conditions).**

During a travel activity, the variable message signs are used to provide travel related information in real time. Each type of drivers has two route choice strategies: *S*_1_: choose the route of the shortest route (route *A*); *S*_2_: choose the recommended route (route *B*).

According to the difference of individual preference, we assume that participant *T*_1_ consisted of the rigid demand drivers and participant *T*_2_ is composed of the flexible demand drivers. *T*_1_ and *T*_2_ play game in this transportation system; the object of them is the individual utility maximization for each other. During the game, the choice result is not determined in advance but changes as the study process and the driver's strategy adjustment due to their experiences and the real-time traffic information [31].

The payoff of each participant under different decision conditions is represented as follows:

- *T*_1_ chooses route *A* while *T*_2_ chooses route *A*: the payoff of  *T*_1_ is *V*_1(*A*)_ − *R*, and the payoff of  *T*_2_ is *V*_2(*A*)_ − *R*;
- *T*_1_ chooses route *A* while *T*_2_ chooses route *B*: the payoff of  *T*_1_ is *V*_1(*A*)_ + *R*_1_, and the payoff of  *T*_2_ is *V*_2(*B*)_ − *D*_1_;
- *T*_1_ chooses route *B* while *T*_2_ chooses route *A*: the payoff of  *T*_1_ is *V*_1(*B*)_ − *D*_2_, and the payoff of  *T*_2_ is *V*_2(*A*)_ + *R*_2_;
- *T*_1_ chooses route *B* while *T*_2_ chooses route *B*: the payoff of  *T*_1_ is*V*_1(*B*)_ − *D*_3_, and the payoff of  *T*_2_ is *V*_2(*B*)_ − *D*_4_.

Table 1 is the payoff matrix.

When the two participants choose different routes, the participant who chooses route *A* will benefit from the good traffic condition while the participant choosing route *B* will get losses because of the increased detouring distance. Considering the rigid demand of  *T*_1_, we can conclude that *R*_1_ > *R*_2_ and *D*_2_ > *D*_1_. If all the participants choose route *B*, because of the individual preference difference, the utility reduced degree of *T*_1_ is bigger than the utility reduced degree caused by the case that *T*_1_ chooses route *B* and *T*_2_ chooses route *A*; that is, *D*_3_ > *D*_2_. Similarly, the conclusion that *D*_4_ > *D*_1_ is drawn. Moreover, the utility reduced degree of *T*_1_ from route *A* to route *B* is bigger than that of *T*_2_, that is *D*_2_ > *D*_4_. According to the above analysis, it can be summarized that *D*_3_ > *D*_2_ > *D*_4_ > *D*_1_ and *R*_1_ > *R*_2_.

**Step 3 (construct the route choice model).**

Assume that the probabilities of choosing route *B* of *T*_1_ and *T*_2_ are *x* and *y*  (*x*, *y* ∈ [0,1]), respectively. Accordingly, the probabilities of choosing route *A* are 1 − *x* and 1 − *y*, respectively.

In conclusion, the route choice game model which embeds CPT can be expressed as follows:

(16) $$\begin{array}{c} \text{Player:}\;\;T_{1},T_{2}\\ \text{Strategy}\;\;\text{set:}\;\;\left\{S_{1},S_{2}\right\}\\ \text{Payoff}\;\;\text{matrix:}\;\;\text{see}\;\text{Table}\;1. \end{array}$$

**Step 4 (dynamic evolutionary analysis).**

Section 3.3 will discuss the dynamic evolutionary process in detail.

### 3.3. Dynamic Evolutionary Analysis

The utility of strategy *S*_2_ of  *T*_1_  (*U*_1*B*_) consisted of two parts. The first part is *T*_1_'s utility that *T*_1_ chooses route *B* while *T*_2_ chooses route *A*. The second part is *T*_1_'s utility that *T*_1_ chooses route *B* while *T*_2_ chooses route *B*. The utility of strategy *S*_1_ of  *T*_1_ is obtained by the same principle. *U*_1*B*_ and *U*_1*A*_ are represented as follows:

(17) U1B=y·V1B−D3+1−y·V1B−D2=D2−D3y+V1B−D2,U1A=y·V1A+R1+1−y·V1A−R=R1+Ry+V1A−R.

The average utility of strategies *S*_1_ and *S*_2_ of  *T*_1_ is the average utility of *S*_1_ and *S*_2_. The former utility equals the selected proportion of *S*_1_ multiplies the utility of *S*_1_. The latter is the selected proportion of *S*_2_ multiplies the corresponding utility. For the sake of convenience in the process of discussion, *V*_*iA*_ = *V*_*iB*_  (*i* = 1,2) is assumed. Then, the average utility of strategies *S*_1_ and *S*_2_ of  *T*_1_ is expressed as follows:

(18) U−1=x·U1B+1−x·U1A=D2−R−R1−D3xy+R−D2x +R1+Ry+V1A−R.

In evolutionary game theory, the dynamic change rate of strategy proportion is the core of the bounded rational game analysis. The change rate depends on the player's learning ability and learning rate. This process can be represented by the replicator dynamics. The replicator dynamics of strategy *S*_2_ to participant *T*_1_ is

(19) dxdt=x·U1B−U−1=x1−x ×D2−R−R1−D3y+R−D2.

To participant *T*_2_, the utility of strategy *S*_2_ (*U*_1*B*_) and strategy *S*_1_ (*U*_1*A*_) is expressed as below, respectively:

(20) U2B=x·V2B−D4+1−x·V2B−D1=D1−D4x+V2B−D1,U2A=x·V2A+R2+1−x·V2A−R=R2+Rx+V2A−R.

The average utility of strategies *S*_1_ and *S*_2_ of  *T*_2_ is as follows:

(21) U−2=y·U2B+1−y·U2A=D1−R−R2−D4xy+R2+Rx +R−D1y+V2A−R.

The replicator dynamics of strategy *S*_2_ of  *T*_2_ is

(22) dydt=y·U2B−U−2=y1−yD1−R−R2−D4x+R−D1.

A fixed point of the replicator dynamics is a population that satisfies ${\dot{x}}_{i}=0$, ∀*i*. Fixed point describes the situation that there is no longer evolution. The fixed points of this route choice system are (0,0), (0,1), (1,0), (1,1), and ((*D*_1_ − *R*)/(*D*_1_ − *R* − *R*_2_ − *R*_4_), (*D*_2_ − *R*)/(*D*_2_ − *R* − *R*_1_ − *D*_3_)). We utilize Jacobin matrix to discuss the ESS under different evolution paths.

The Jacobin matrix is

(23) $$\begin{array}{c} J=\left[\begin{array}{c} \left(1-2x\right)\left[\left(D_{2}-R-R_{1}-D_{3}\right)y+\left(R-D_{2}\right)\right],x\left(1-x\right)\left(D_{2}-R-R_{1}-D_{3}\right)\\ y\left(1-y\right)\left(D_{1}-R-R_{2}-D_{4}\right),\left(1-2y\right)\left[\left(D_{1}-R-R_{2}-D_{4}\right)x+\left(R-D_{1}\right)\right] \end{array}\right]. \end{array}$$

The determinant of the Jacobin matrix is

(24) det⁡J=1−2xD2−R−R1−D3y+R−D2 ×1−2yD1−R−R2−D4x+R−D1 −x1−xD2−R−R1−D3y1−y ×D1−R−R2−D4.

The trace of the Jacobin matrix is

(25) tra J=1−2xD2−R−R1−D3y+R−D2 +1−2yD1−R−R2−D4x+R−D1.

(*1) R* = 0. The practical meaning of *R* = 0 is that there is no installation of VMS in the transportation system. There are 4 equilibrium points under this scenario, and they are (0,0)  (0,1)  (1,0)  (1,1). The evolutionary equilibrium analysis result is illustrated in Table 2.

From Table 2 it can be seen that there is a strictly dominant pure strategy (0,0), so it is the ESS. The ESS means that, in the long run, the system will tend to the evolutionary stable state that the proportion of strategy *S*_2_ of  *T*_1_ and *T*_2_ is *x* = 0, *y* = 0, and the stable state will not be disturbed by a small portion of mutation. In other words, all the drivers will choose the shortest route (route *A*) when they cannot get more information about the alternative routes in the transportation system.

(*2) *0 < *R* < *D*_1_. The practical meaning of 0 < *R* < *D*_1_ is that the travel efficiency loss caused by the congestion that all of the drivers choose route *A* is small enough. The equilibrium points are (0,0)  (0,1)  (1,0)  (1,1).

From the evolutionary equilibrium analysis (Table 3), (0,0) is the strictly dominant pure strategy. It suggests that when the travel efficiency losses are small, all the drivers will choose route *A*, and the transportation system will progress toward the evolutionary stable state that the proportion of  *T*_1_ and *T*_2_ selecting route *B* is *x* = 0 and *y* = 0.

(*3) D*_1_ < *R* < *D*_2_. The practical meaning of *D*_1_ < *R* < *D*_2_ is that the travel efficiency loss shown on VMS is between *D*_1_ and *D*_2_. In this scenario, (0,0)  (0,1)  (1,0)  (1,1) are the equilibrium points. The evolutionary equilibrium result is shown in Table 4.

Table 4 shows that the system with equilibrium point (0,1) is local stable, and this strategy is ESS of this dynamic route choice system. Under the influence of information, the system will develop towards the evolutionary stable state that *T*_1_ chooses route *A* while *T*_2_ chooses route *B*.

(*4) R* > *D*_2_. The practical meaning of *R* > *D*_2_ is that the travel efficiency loss caused by the congestion of route *A* is great. There are four equilibrium points under this scenario, and they are (0,0)  (0,1)  (1,0)  (1,1). The evolutionary equilibrium analysis result is illustrated in Table 5.

From Table 5, the following conclusion can be drawn that the dynamic system has two pure strategies, and they are (0,1) and (1,0). The two equilibrium points are both ESS. It means that when the drivers in the system get the information relevant to the great losses, the system state will progress towards the evolutionary stable state that *x* = 0 and *y* = 1 or *x* = 1, *y* = 0. That is to say, under the influence of traffic information, one of the participants will switch to route *B*, while the other driver will persist in the choice of route *A*. The dynamic system will progress to stable state which is related to the initial value of the payoff matrix.

The above uncertainty can be solved by the stability theorem. The stability theorem of differential equations to distinguish the different stable states can be expressed in mathematics: ∀*x* > *x* ^*^, considering that *F*(*x*) = *dx*/*dt* < 0, that is, *F*′(*x* ^*^) < 0, thus the system is stable on the point *x* ^*^.

To participant *T*_1_, there are two equilibrium points in the replicator dynamics, *x* ^*^ = 0, *x* ^*^ = 1. Let $F\left(x\right)=dx/dt=x\cdot (U_{1B}-{\overset{-}{U}}_{1})=x(1-x)[(D_{2}-R-R_{1}-D_{3})y+(R-D_{2})]$; then *F*′(*x*) = (1 − 2*x*)[(*D*_2_ − *R* − *R*_1_ − *D*_3_)*y* + (*R* − *D*_2_)].

Take *x* ^*^ = 0 and *x* ^*^ = 1 into *F*′(*x*) and judge the stability of the system at the equilibrium point according to the result of  *F*′(*x* ^*^). The proportion of  *T*_1_ choosing route *A* will eventually progress to different stable states depending on the different initial values of route selection proportion of  *T*_2_.

When *y* = (*D*_2_ − *R*)/(*D*_2_ − *R* − *R*_1_ − *D*_3_), *F*′(*x*) ≡ 0. It reveals that no matter what the initial proportion of *T*_1_ choosing rout *B* is, the system is stable.

When 0 < *y* < (*D*_2_ − *R*)/(*D*_2_ − *R* − *R*_1_ − *D*_3_),  *F*′(0) > 0, *F*′(1) < 0, and the stable point of the system is *x* ^*^ = 1. It means that the proportion of choosing route *B* of  *T*_1_ will be stable at 100% as time changes.

When (*D*_2_ − *R*)/(*D*_2_ − *R* − *R*_1_ − *D*_3_) < *y* < 1, *F*′(0) < 0, *F*′(1) > 0, and the system stable point is *x* ^*^ = 0. It indicates that participant *T*_1_ will switch to route *A* as time goes on.

**Figure 5:**
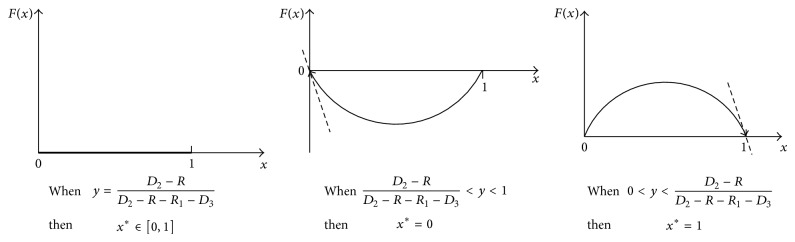
Replicated dynamic phase of participant *T*_1_.

The group replicated dynamic phase of  *T*_1_ is exhibited in Figure 5.

To participant *T*_2_, there are two equilibrium points in the replicator dynamics, *y* ^*^ = 0 and *y* ^*^ = 1. Let $F\left(y\right)=dy/dt=y\cdot \left(U_{1B}-{\overset{-}{U}}_{1}\right)=y\left(1-y\right)\left[\left(D_{1}-R-R_{2}-D_{4}\right)y+\left(R-D_{1}\right)\right]$; then *F*′(*y*) = (1 − 2*y*)[(*D*_1_ − *R* − *R*_2_ − *D*_4_)*y* + (*R* − *D*_1_)].

**Figure 6:**
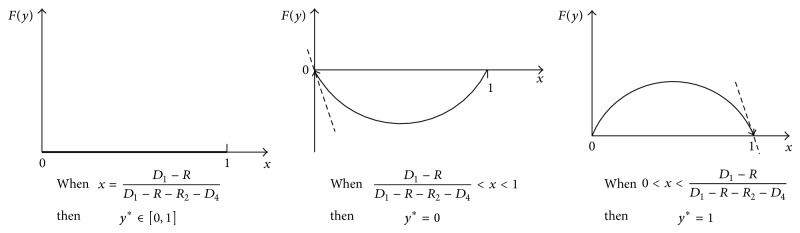
Replicated dynamic phase of participant *T*_2_.

Take *y* ^*^ = 0 and *y* ^*^ = 1 into *F*′(*y*); the changing process of *y* is analyzed in Figure 6.

When *x* = (*D*_1_ − *R*)/(*D*_1_ − *R* − *R*_2_ − *D*_4_), *F*′(*y*) ≡ 0. It indicates that whatever the initial proportion of *T*_2_ choosing route *B* is, the dynamic transportation system is stable.

When 0 < *x* < (*D*_1_ − *R*)/(*D*_1_ − *R* − *R*_2_ − *D*_4_), *F*′(0) > 0, *F*′(1) < 0, and the system stable point is *y* ^*^ = 1. It reveals the proportion that participant *T*_2_ choosing route *B* will increase to 100% as time goes by.

When (*D*_1_ − *R*)/(*D*_1_ − *R* − *R*_2_ − *D*_4_) < *x* < 1, *F*′(0) < 0, *F*′(1) > 0, and the stable point of the system is *y* ^*^ = 0. It means that participant *T*_2_ will switch to route *A* as time changes.

**Figure 7:**
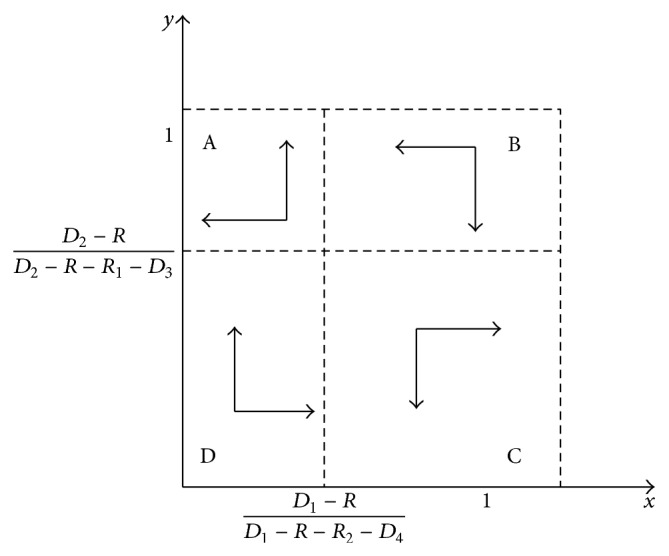
Group replicated dynamic phase of  *T*_1_ and *T*_2_.

The stability of groups *T*_1_ and *T*_2_ is illustrated in Figure 7.

When the initial state of *x* and *y* is in region A, ESS is *x* ^*^ = 0, *y* ^*^ = 1, and the dynamic transportation system will evolve towards the stable state that *T*_1_ chooses route *A* while *T*_2_ chooses route *B*. When the initial state is in region C, ESS is *x* ^*^ = 1, *y* ^*^ = 0, and the system will stabilize at the state that *T*_1_ chooses route *B* while *T*_2_ chooses route *A* finally. When the initial state is in regions B and D, the direction of evolution is uncertain. It may evolve to region A and converge to (0,1) or evolve to region C and converge to (1,0).

## 4. Discussion and Conclusion

This paper has embedded cumulative prospect theory into evolutionary game theory in order to integrate the individual perception and decision schemes with the group learning and evolutions. This paper discussed the drivers' route choice behaviors and the corresponding stable state of the dynamic traffic system according to the different information shown on VMS.

When there is no VMS in the transportation system (*R* = 0), all the drivers choose the shortest route (route *A*). When the travel efficiency losses displayed on VMS are small enough (0 < *R* < *D*_1_), the impact of VMS on route choice is indistinctive. The result of the evolution analysis turns out to be that all the drivers still choose the shortest route. When the travel efficiency losses shown on VMS are appropriate (*D*_1_ < *R* < *D*_2_), the transportation system progresses to the evolutionary stable state, in which the drivers with rigid demand choose route *A* while the drivers with flexible demand choose route *B*. When the travel efficiency losses value is big enough (*R* > *D*_2_), the analytic result suggests that the drivers are sensitive to the efficiency losses, and the transportation system progresses towards the evolutionary stable state that one type of the drivers chooses route *A* and the other type chooses route *B*. Our findings indicate that the stable state of the dynamic route choice system is sensitive both to the traffic information and to the initial state of the transportation system.

However, there are some limitations in this research. First, the modeling method presented here is effective but needs to be validated in the empirical work. Another issue is that our findings are valid only for the assumption that the distribution of driver's characteristic is identical in the same participant.

We suggest that the survey data should be collected in order to calibrate the parameters of the proposed model and to investigate the capability of the model to explain the field observations. In the future, an investigation on the effect of the drivers with different characteristic distributions should be carried out.

The results of this study may be useful to learn the driver's route choice behavior and to alleviate the urban traffic congestion. The potential applications of the proposed method involve the modeling and describing the group choice evolution process from the perspective of the individual risk attitude as well as the decision-making schemes. It is suitable for capturing the adaptation course of the group choice.

## Figures and Tables

**Table 1:** Payoff matrix under different decision conditions.

*T*_1_	*T*_2_	
	Route *A*	Route *B*
Route *A*	*V*_1__(*A*)_ − *R*, *V*_2__(*A*)_ − *R*	*V*_1__(*A*)_ + *R*_1_, *V*_2__(*B*)_ − *D*_1_
Route *B*	*V*_1__(*B*)_ − *D*_2_, *V*_2__(*A*)_ + *R*_2_	*V*_1__(*B*)_ − *D*_3_, *V*_2__(*B*)_ − *D*_4_

**Table 2:** Local stability analysis.

Equilibrium point	det **J**	Sign of det **J**	tra **J**	Sign of tra **J**	Local stability
(0,0)	*D*_1_ *D*_2_	+	−(*D*_1_ + *D*_2_)	−	ESS
(0,1)	−*D*_1_(*D*_3_ + *R*_1_)	−	*D*_1_ − *D*_3_ − *R*_1_	−	Instability
(1,0)	−*D*_2_(*D*_4_ + *R*_2_)	−	*D*_2_ − *D*_4_ − *R*_2_	−	Instability
(1,1)	(*R*_1_ + *D*_3_)(*R*_2_ + *D*_4_)	+	*R*_1_ + *D*_3_ + *R*_2_ + *D*_4_	+	Instability

**Table 3:** Local stability analysis.

Equilibrium point	det **J**	Sign of det **J**	tra **J**	Sign of tra **J**	Local stability
(0,0)	(*R* − *D*_2_)(*R* − *D*_1_)	+	(*R* − *D*_2_) + (*R* − *D*_1_)	−	ESS
(0,1)	(*R*_1_ + *D*_3_)(*R* − *D*_1_)	−	(*D*_1_ − *D*_3_) − (*R* + *R*_1_)	−	Instability
(1,0)	(*R* − *D*_2_)(*R*_2_ + *D*_4_)	−	*D*_2_ − *D*_4_ − *R* − *R*_2_		Instability
(1,1)	(*R*_1_ + *D*_3_)(*R*_2_ + *D*_4_)	+	*R*_1_ + *D*_3_ + *R*_2_ + *D*_4_	+	Instability

**Table 4:** Local stability analysis.

Equilibrium point	det **J**	Sign of det **J**	tra **J**	Sign of tra **J**	Local stability
(0,0)	(*R* − *D*_2_)(*R* − *D*_1_)	−	(*R* − *D*_2_) + (*R* − *D*_1_)		Instability
(0,1)	(*R*_1_ + *D*_3_)(*R* − *D*_1_)	+	(*D*_1_ − *D*_3_) − (*R* + *R*_1_)	−	ESS
(1,0)	(*R* − *D*_2_)(*R*_2_ + *D*_4_)	−	*D*_2_ − *D*_4_ − *R* − *R*_2_		Instability
(1,1)	(*R*_1_ + *D*_3_)(*R*_2_ + *D*_4_)	+	*R*_1_ + *D*_3_ + *R*_2_ + *D*_4_	+	Instability

**Table 5:** Local stability analysis.

Equilibrium point	det **J**	Sign of det **J**	tra **J**	Sign of tra **J**	Local stability
(0,0)	(*R* − *D*_2_)(*R* − *D*_1_)	+	(*R* − *D*_2_) + (*R* − *D*_1_)	+	Instability
(0,1)	(*R*_1_ + *D*_3_)(*R* − *D*_1_)	+	(*D*_1_ − *D*_3_) − (*R* + *R*_1_)	−	ESS
(1,0)	(*R* − *D*_2_)(*R*_2_ + *D*_4_)	+	*D*_2_ − *D*_4_ − *R* − *R*_2_	−	ESS
(1,1)	(*R*_1_ + *D*_3_)(*R*_2_ + *D*_4_)	+	*R*_1_ + *D*_3_ + *R*_2_ + *D*_4_	+	Instability
